# Microscopically-Tuned Band Structure of Epitaxial Graphene through Interface and Stacking Variations Using Si Substrate Microfabrication

**DOI:** 10.1038/srep05173

**Published:** 2014-06-06

**Authors:** Hirokazu Fukidome, Takayuki Ide, Yusuke Kawai, Toshihiro Shinohara, Naoka Nagamura, Koji Horiba, Masato Kotsugi, Takuo Ohkochi, Toyohiko Kinoshita, Hiroshi Kumighashira, Masaharu Oshima, Maki Suemitsu

**Affiliations:** 1 Research Institute of Electrical Communication, Tohoku University, 2-1-1 Katahira, Aobaku-ku, Sendai, Miyagi 980-8577, Japan; 2 Core Research for Evolutional Science and Technology, Japan Science and Technology Agency, 5-7, Goban-cho, Chiyoda-ku, Tokyo 102-0076, Japan; 3 School of Engineering, Tohoku University, 6-6-01, Aramaki, Aoba-ku, Sendai 980-8578, Japan; 4 Department of Applied Chemistry, Graduate School of Engineering, The University of Tokyo, 7-3-1 Hongo, Tokyo 113-8656, Japan; 5 Synchrotron Radiation Research Organization, The University of Tokyo, 1-1-1 Kouto, Sayo-cho, Sayo-gun, Hyogo 679-5198, Japan; 6 JASRI/SPring-8, 1-1-1 Kouto, Sayo, Hyogo 679-5198, Japan; 7 Photon Factory, Institute of Materials Structure Science, High Energy Accelerator Research Organization, Ibaraki 305-0801, Japan

## Abstract

Graphene exhibits unusual electronic properties, caused by a linear band structure near the Dirac point. This band structure is determined by the stacking sequence in graphene multilayers. Here we present a novel method of microscopically controlling the band structure. This is achieved by epitaxy of graphene on 3C-SiC(111) and 3C-SiC(100) thin films grown on a 3D microfabricated Si(100) substrate (3D-GOS (graphene on silicon)) by anisotropic etching, which produces Si(111) microfacets as well as major Si(100) microterraces. We show that tuning of the interface between the graphene and the 3C-SiC microfacets enables microscopic control of stacking and ultimately of the band structure of 3D-GOS, which is typified by the selective emergence of semiconducting and metallic behaviours on the (111) and (100) portions, respectively. The use of 3D-GOS is thus effective in microscopically unlocking various potentials of graphene depending on the application target, such as electronic or photonic devices.

Carrier dynamics in monolayer graphene, a honeycomb lattice consisting of carbon atoms, are described in terms of quantum electrodynamics^1^,^2^,^3^,^4^, as a consequence of the linear band dispersion relation. Graphene therefore possesses excellent electronic properties, such as giant carrier mobilities. In addition, multilayer graphene has the unique feature that its electronic properties, e.g., the linearity of the band dispersion, are tunable by changing the stacking sequence^5^. Graphene is a promising material for various next-generation device applications, such as high-electron-mobility transistors (HEMTs)^6^,^7^, saturable absorbers^8^,^9^ and terahertz lasers^10^.

One of the major challenges to such device applications of graphene is that of establishing epitaxial growth of graphene on practical substrates using methods compatible with existing device technologies^7^. The chemical vapor deposition method (CVD) produces large-scale, high-quality graphene^11^,^12^, but it also requires that a transfer process be performed to move the graphene from a metal substrate to an insulating substrate, during which it is hard to completely avoid metal contamination^12^. Epitaxial growth of graphene on SiC bulk crystals also produces large-scale, high-quality graphene on SiC substrates, which are already in use for power device applications^13^,^14^. Epitaxial graphene (EG) has, however, a major drawback: the high production cost of the wafer. To overcome this drawback, epitaxial growth of graphene on Si (GOS) substrates by sublimating surface silicon atoms of 3C-SiC thin films on the Si substrates at elevated temperatures has been developed, up to the wafer scale^15^,^16^,^17^,^18^. Formation of graphene has been confirmed by Raman spectroscopy^15^,^16^,^17^,^18^, low-energy electron diffraction (LEED)^17^,^18^,^19^,^20^ and photoelectron spectroscopy (PES)^19^,^20^. Despite the mediocre quality of initial GOS, recent improvements in GOS technology now provide a material that clearly demonstrates the linear dispersion of the π band near the Dirac point by angle-resolved PES^21^. The potential of GOS for electronic and photonic device applications has been demonstrated by fabricating transistors^22^,^23^, terahertz photonic devices^24^ and even logic inverters^25^, a fundamental component for integrated circuits. GOS is thus becoming a promising graphene production method, based on its advantages, such as the cost of the wafer and compatibility with existing Si-based device technologies.

In addition to these manufacturing advantages, GOS has another more profound advantage: the tunability of the band structure by altering the Si substrate. By controlling the crystallographic orientation of the Si substrate, we can select the surface orientation of the 3C-SiC thin film, which eventually makes it possible to modulate the stacking of graphene and its interface structure with SiC. The latter controls the presence or absence of the interfacial (6√3 × 6√3)-reconstructed buffer layer, which is a precursor for graphene in EG and GOS. On the Si(111) substrate, the buffer layer is present, and GOS is Bernal-stacked^17^,^18^; the energy band then loses its linearity. It is, however, multiply-split, and a bandgap can be opened, which is suitable for electronic device applications. On the other hand, on Si(110) and Si(100) substrates, the buffer layer is absent, and GOS is not Bernal-stacked^17^,^18^,^20^. The GOS on Si(110) forms turbostratic stacks^20^, while GOS on 3C-SiC(100)/Si(100) has rotational stacking faults with a finite angle of 15 degrees^17^. The energy bands in these cases are anticipated to maintain their linear natures; the bands are not split, and the bandgap is closed. This feature is promising for photonic device applications, especially in the terahertz region. Such controllability over physical properties of graphene simply by changing the crystallographic orientation of Si substrates is definitely one of the major advantages of the GOS technology. It is not easy to take a similar strategy by using SiC bulk crystals. If we can vary the crystallographic orientations within an identical Si wafer, however, this would even enrich the applicability of GOS. This is actually possible by use of the well-matured Si microfabrication technology. The questions, then, are (1) whether similar graphene structures are reproduced on such microfabricated, neighboring (100) and (111) portions as on independent wafers, and (2) whether the controllability of the physical properties of graphene is microscopically realized on (100) and (111) portions on a single Si substrate. The question (2) makes sense when we recall the fact that a continous few-layer graphene grows over both the (100) and the (111) portions^21^,^26^.

In this work, we demonstrate the microscopic tuning of the band structure of epitaxial graphene on a 3D microfabricated Si substrate (3D-GOS) through stacking with the aid of microelectromechanical system (MEMS) technology, specifically anisotropic wet etching which produces (111) and (100) microfacets on a Si(100) substrate.

## Results

### 3D-GOS formation

**Figure 1:**
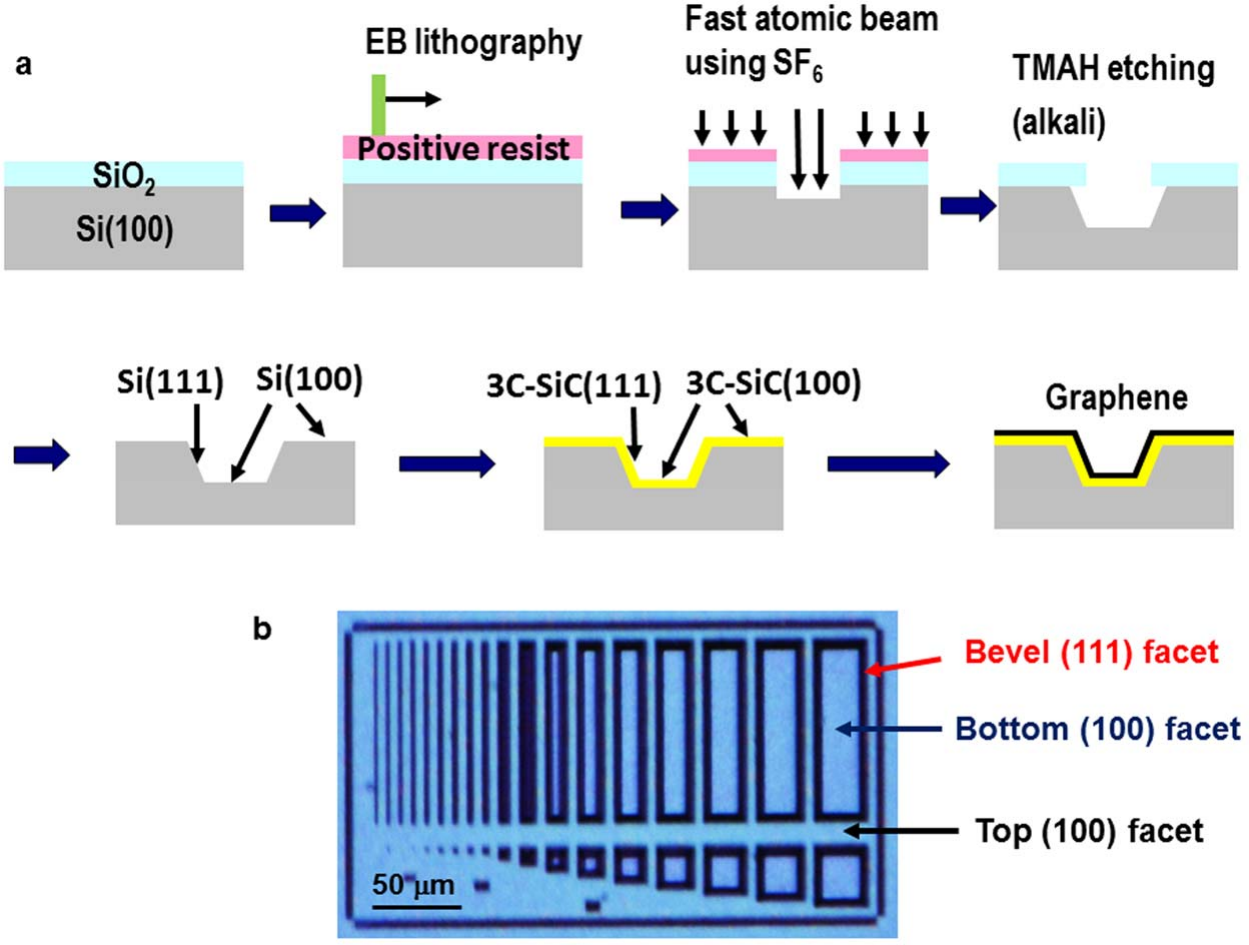
3D-GOS Fabrication. (a) Schematic of the 3D-GOS fabrication procedure. (b) Optical microscope image of the 3D-GOS.

The formation of 3D-GOS is schematically shown in Figure 1. First, a sacrificial SiO_2_ film (90 nm) is grown on a Si(100) substrate (*p*-type, 1–10 Ω cm) by the dry oxidation method. Electron-beam lithography is performed on the sacrificial SiO_2_ thin film, followed by fast atomic beam etching using SF_6_ as an etchant^27^,^28^, to leave behind the SiO_2_ mask on the surface. Using the SiO_2_ mask, the surface is anisotropically etched in a 25% tetramethyl ammonium hydroxide (TMAH) aqueous solution at 353 K for 6 min. The etching by the TMAH aqueous solution is used to expose Si(111) microfacets on Si(100) substrates, which are frequently used as the standard fabrication procedure for MEMS devices^29^. The etching depth is ~1 μm. Si(111) microfacets then form on the Si(100) substrate, followed by the removal of the SiO_2_ mask with a treatment in a 5% HF aqueous solution. The 3C-SiC thin films (~100 nm in thickness) are grown on microfabricated Si(100) substrate by using gas-source molecular beam epitaxy with monomethylsilane as a gas source at a substrate temperature of 1353 K^15^. Due to its almost conformal growth, the 3C-SiC(111) and 3C-SiC(100) microfacets are formed thereon. Finally, the substrate is annealed *in vacuo* at 1523 K for 30 min to grow graphene epitaxially on the surface of the 3C-SiC(111) microfacet^15^,^16^,^17^,^18^,^27^. Various kinds of micropatterns (Fig. 1b) are formed on the sample to calibrate the device fabrication for future fabrication projects.

### Microscopic variation of the interface of 3D-GOS

**Figure 2:**
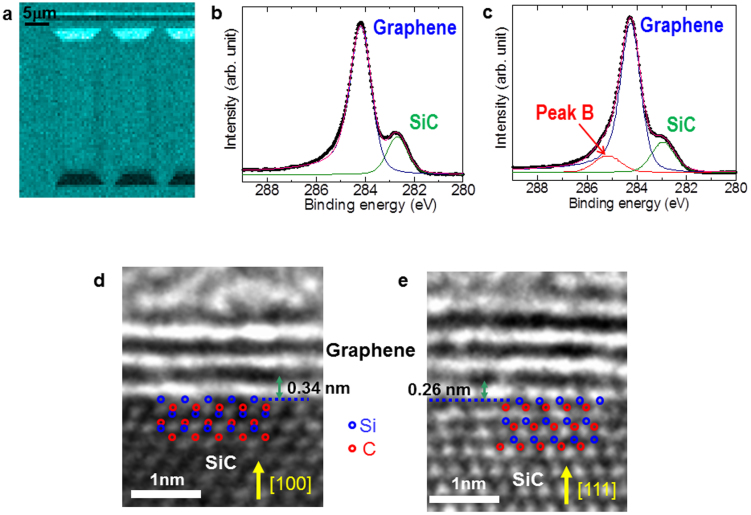
Interface analysis of 3D-GOS. (a) Intensity mapping of the 3D-GOS by using C1s core-level photoelectrons (~284 eV). (b) Pinpoint C1s core-level spectrum of graphene on the 3C-SiC(100) microterrace. (c) Pinpoint C1s core-level spectrum of graphene on the 3C-SiC(111) microfacet. (d) X-TEM image of the interface between graphene and the 3C-SiC(100) microterrace. (e) X-TEM image of the interface between graphene and the 3C-SiC(111) microfacet.

The interface between graphene and the 3C-SiC microfacets is examined here because the graphene growth mode is determined by the presence or absence of the buffer layer because it works as the template for the Bernal stacking of graphene^17^,^18^,^20^. The interface chemical composition is probed nanoscopically by using three-dimensional high-resolution scanning photoelectron microscopy (3D nano-ESCA (electron spectroscopy for chemical analysis)) using a focused incident X-ray beam with a diameter of 70 nm and a photon energy of 1000 eV^30^,^31^. The energy resolution of the spectrometer was set to 300 meV^32^. Figure 2a shows the photoelectron intensity mapping of the C1s core level (~284 eV) due to the graphene of 3D-GOS. The sample surface is found to be covered with graphene on both the (100) and (111) facets, although the image contrast reflecting the microfabrication pattern of 3D-GOS arises from the difference in relative angle of the microfacets to the detector used for 3D nano-ESCA. Figure 2b,c shows pinpoint C1s core-level photoelectron spectra of graphene on the 3C-SiC(100) and 3C-SiC(111) microfacets using 3D nano-ESCA. The peaks due to the 3C-SiC thin films (~283 eV)^17^,^19^ and graphene (~284 eV)^17^,^19^ are visible in both of the spectra. The ratio of the intensity of the SiC peak to the intensity of the graphene peak is decreased by increasing the photoelectron emission angle from the surface normal (surface-sensitive mode), as seen in the angle-resolved spectra (see Fig. S1). This confirms that graphene is present on the 3C-SiC microfacets. The estimated layer numbers of graphene on the 3C-SiC(100) and 3C-SiC(111) microfacets are 2.7 and 2.4, respectively, using the standard equation using the intensity ratios of the graphene and SiC peaks (see Supplementary Note 1). The difference between the spectra is the presence of the shoulder in the spectrum of graphene on the 3C-SiC(111) microfacet, labelled as peak B at the higher binding energy (~285 eV). The analysis of the angle-resolved spectra clarifies that peak B is ascribable to the buffer layer situated between the graphene and the 3C-SiC(111) microfacet^13^,^17^ (see Supplementary Note 2). The buffer layer is thus formed selectively between the graphene and the 3C-SiC(111) microfacet. A reason for this selective formation is that the buffer layer is commensurate with the 3C-SiC(111) surface and energetically favorable^33^,^34^, while the twofold symmetry of the 3C-SiC(100) surface may inhibit the formation of such a buffer layer.

The atomic structure of the interfaces between graphene and the 3C-SiC microfacets has been investigated by using cross-sectional transmission electron microscopy (X-TEM), as shown in Figure 2d,e. Planar-shaped graphene and 3C-SiC are atomically resolved in both of the X-TEM images. The interlayer distance between the graphene layers is 0.34 ± 0.07 nm, which agrees well with the interlayer distance of graphite as well as that of GOS using flat Si substrates^29^. The layer numbers (2 to 3) obtained from the X-TEM images are consistent with those estimated from the pinpoint spectra. The difference is the distance between the nearest layer and the 3C-SiC topmost layer. The distance (0.26 ± 0.07 nm) between the nearest layer (buffer layer) and the 3C-SiC(111) microfacet is shorter than that between the nearest layer (graphene) and 3C-SiC(100) (0.34 ± 0.07 nm). This shortened distance is explained by the presence of covalent bonds in the buffer layer that are tied to the surface of the 3C-SiC(111) microfacet^35^. No such covalent bonds exist between the nearest graphene layer and the surface of the 3C-SiC(100) microfacet^35^. The X-TEM observation corroborates the 3D nano-ESCA measurement which shows that the interface varies with the crystallographic orientation of 3C-SiC microfacets grown on the microfabricated Si(100) substrates.

### Microscopic variation of the stacking of 3D-GOS

**Figure 3:**
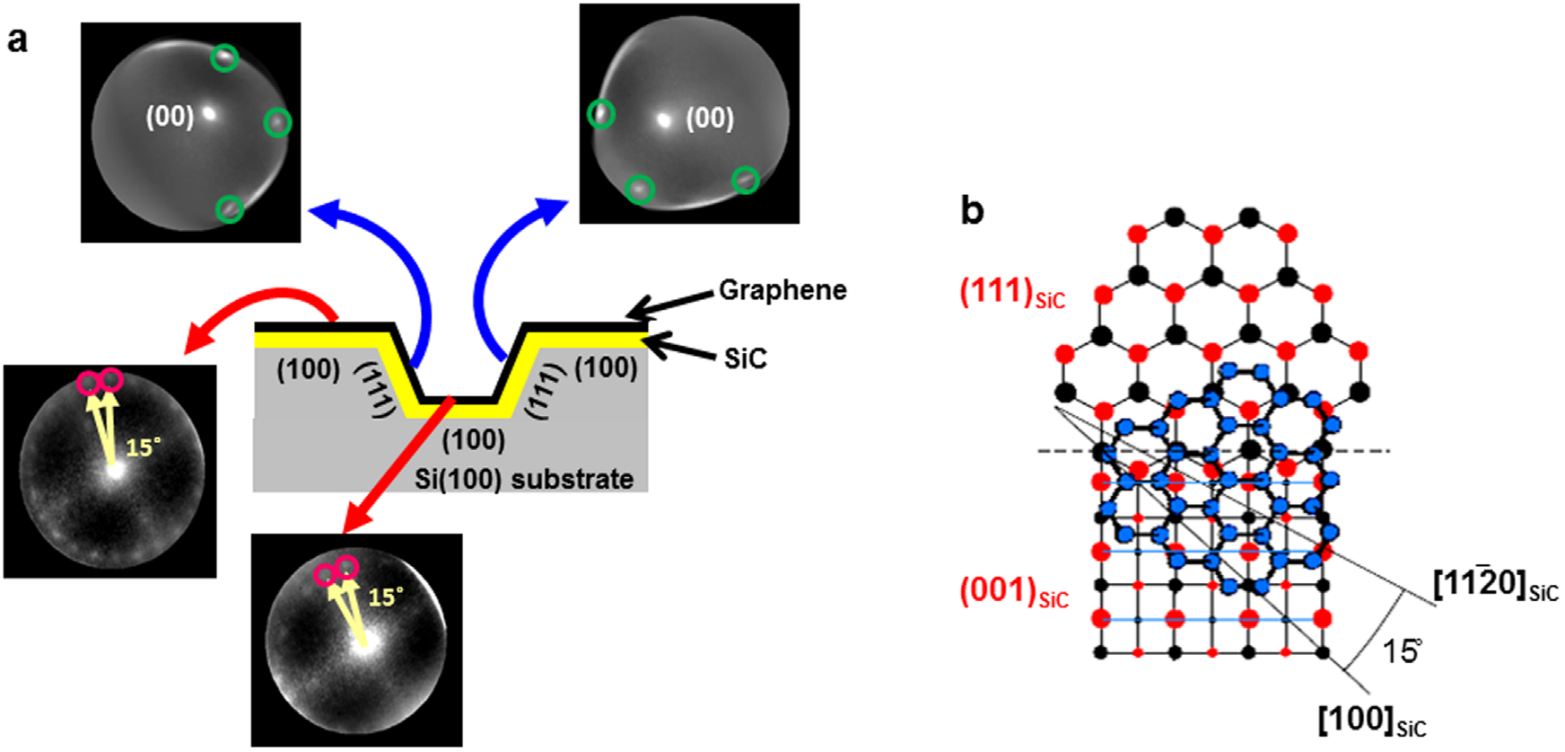
Stacking variation of 3D-GOS. μ-LEED analysis of 3D-GOS from the regions of the top (100) microterrace, bottom (100) microterrace and (111) bevel. The green circles indicate (1 × 1) spots from the Bernal-stacked graphene. The pink circles indicate the adjacent spots from rotationally-stacked graphene. The energy of the incident electrons is 50 eV of the μ-LEED observation.

To clarify the relation of the observed interface variation caused by microfacets with the graphene stacking, microscopic LEED (μ-LEED) analysis is performed for 3D-GOS, as shown in Figure 3. The LEED images were taken by collecting diffracted electrons from selected areas of 1-μm diameter^17^,^18^,^20^. The distortion of the μ-LEED images is due to the inhomogeneous surface electric field arising from the inclination of the (111) microfacets. This is because the diffracted electrons are having lower kinetic energies (~50 eV) near the surface and are thus susceptible to the inhomogeneity of the surface electric field^17^.

As seen from the μ-LEED images of the two bevels, a hexagonal LEED pattern from graphene is clearly identified on the 3C-SiC(111) microfacets. The spots due to the specular reflection, labelled as (00) in the images, are located away from the center of the μ-LEED pattern because of the inclination of the 3C-SiC(111) microfacets. The μ-LEED pattern is almost the same as that of the graphene on the flat Si(111) substrate and graphene on 6H-SiC(0001)^17^,^18^,^20^, which indicates the Bernal stacking of graphene on the 3C-SiC(111) microfacet, although there can be a slight imperfection in the stacking sequence^36^,^37^. In the μ-LEED images, the spots due to the 6√3 × 6√3-reconstructed buffer layer are hardly visible. This can be related to the existence of the graphene overlayers (two to three atomic layer thick) on the buffer layer, which could substantially weaken the diffraction spots from the buffer layer^19^ because of the short escape depth (<1 nm) of the diffracted electrons having low kinetic energies of 50 eV^38^.

In contrast to epitaxial graphene on the 3C-SiC(111) microfacet, the LEED spots of graphene on both the top and bottom 3C-SiC(100) microterraces are rotated, as observed on the graphene on 3C-SiC(100) thin films on flat Si(100) substrates. The rotation angle between the adjacent spots indicated by the yellow arrows is about 15 degrees. One of the possible mechanisms for this rotated growth of graphene on the 3C-SiC(100) face is the {111} facet-induced mechanism^26^. In this mechanism, graphene growth on 3C-SiC(100) substrate is initiated epitaxially at the Si-terminated 3C-SiC(111) microfacets, and the graphene extends over in a carpet-like manner toward the 3C-SiC(100) terrace. This mechanism accounts for the absence of the buffer layer beneath the graphene on 3C-SiC(100)^26^ and the 15 degrees between the spots as well. The graphene's principal axis of 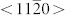 is 15 degrees rotated from the <010> axis of the 3C-SiC(100) terrace in this mechanism (Fig. 3(b)). There are four 3C-SiC{111} microfacets surrounding the 3C-SiC(100) microterrace. Only two out of the four, however, are Si-terminated. The other two are C-terminated, which results in turbostratic stacking and a ring-like LEED pattern^14^,^20^. To explain the 24 spots using this model, therefore, we should consider presence of rotated domains, most probably of anti-phase domains. It is thus demonstrated for 3D-GOS that the stacking varies microscopically in accordance with the variation of the interface structure between graphene and the 3C-SiC microfacet and microterrace.

### Microscopic control of the band structure of 3D-GOS

**Figure 4:**
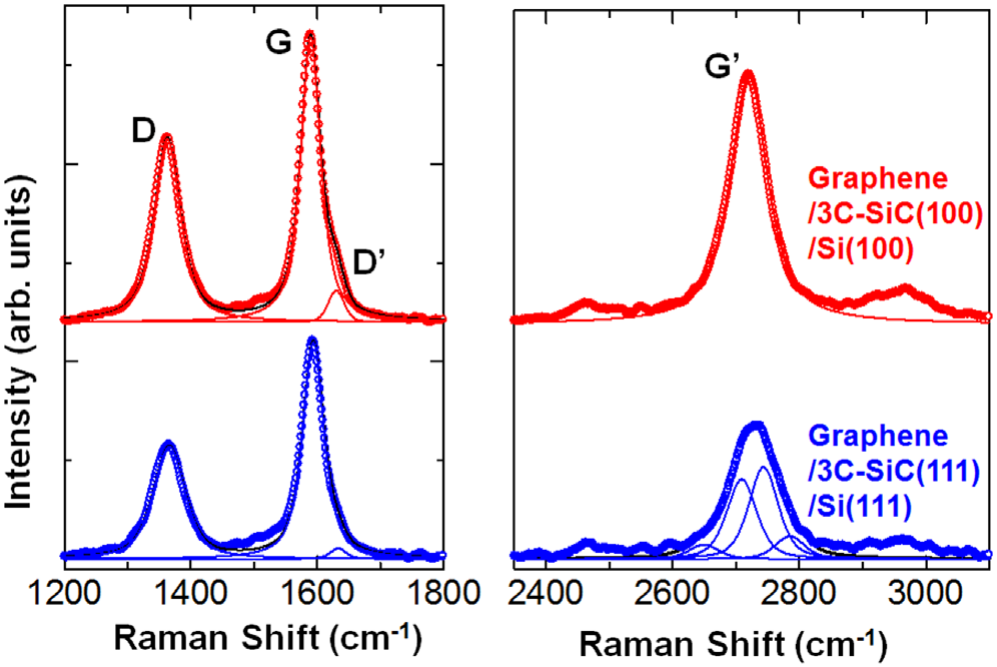
Band structure variation of 3D-GOS. Raman microscopy from graphene on the 3C-SiC(100) microterrace (top) and the 3C-SiC(111) microfacet (bottom). The thin lines indicate the decomposed peak for the G′ bands. The black line in the spectrum of graphene on the 3C-SiC(111) microfacet indicates the synthesized curve.

The band structure of 3D-GOS is anticipated to vary with the microfacet and the microterrace orientation owing to a high susceptibility of the band structure to the stacking sequence, by analogy with the susceptibility of the band structure of GOS using flat Si substrates for stacking depending on the crystallographic orientation of the Si substrate^17^,^20^. To provide proof of this assumption, the band structure of 3D-GOS is microscopically investigated by Raman microscopy, as shown in Figure 4. The fundamental vibration modes of graphene, G (~1600 cm^−1^), D (~1360 cm^−1^), and G′ (~2700 cm^−1^) bands, are visible in the spectra on the graphene on both 3C-SiC(100) microterraces and 3C-SiC(111) microfacets. The G band is the band originating from a single-resonant Raman process at the Γ point^39^. The D band comes from double-resonant Raman processes, and is associated with the presence of defects^39^. The appearance of the D band therefore indicates the existence of defects in graphene on the microfacets and microterraces. The G′ band results from double-resonant processes involving strong electron–phonon coupling^39^,^40^. The G′ band is the overtone of the D band, but is not associated with the presence of defects; rather it is associated with the band structure of graphene^39^. The appearance of these modes corroborates the formation of graphene on the microfacets and the microterraces.

Among these bands, the G′ band is analysed in detail to clarify the band dispersion^41^,^42^,^43^,^44^,^45^. The G′ band is a second-order process related to a phonon near the K point in graphene, activated by double-resonance (DR) processes^39^,^40^, which are responsible for its dispersive nature with the excitation energy and which cause a strong dependence of the lineshape of the G′ band to the band structure of graphene^42^,^44^,^45^. As demonstrated in Figure 4, the G′ band of graphene on the 3C-SiC(100) microterrace is not split and can be represented by a single, symmetrical Lorentzian. The full width at half maximum (FWHM) of the G′ band is 70 cm^−1^. This value is larger than that of epitaxial graphene on 6H-SiC(0001) (37 cm^−1^)^13^ because of the presence of the larger number of defects also inferred from the larger D band^41^. The FWHM in this work is smaller than the previously reported value (80 cm^−1^) for graphene on a 3C-SiC(100) thin film on a Si(100) substrate^46^, which is corroborated by the larger D band in the previous work^46^. The non-splitting of the G′ band suggests that the graphene on the 3C-SiC(100) microterrace has a metallic nature^17^,^20^,^44^,^45^. This arises from the negligible interlayer interaction since the graphene on the 3C-SiC(100) microterrace is not Bernal-stacked, as indicated by the μ-LEED (Fig. 3). This non-Bernal stacking is consistent with the absence of the buffer layer between graphene and the 3C-SiC(100) microterrace, which was confirmed by 3D nano-ESCA (Fig. 2).

On the other hand, the G′ band of the graphene on the 3C-SiC(111) microfacet is asymmetrical and is much broader (90 cm^−1^) than that on the 3C-SiC(100) microterrace (70 cm^−1^). These two features can only be accounted for by considering a set of multiple Lorentzian components, as shown in Figure 4. If the G′ band were to be from a single Lorentzian, its FWHM should have been much smaller than what we observe here. This is because the intensity ratio I_D_/I_G_ of the D band to the G band, which is a good measure of the defect density that broadens the G′ band, is substantially smaller in the graphene on 3C-SiC(111) microfacet (I_D_/I_G_ = 0.64 ± 0.04) than that on the 3C-SiC(100) microterrace (0.75 ± 0.04). Thus, the G′ band on 3C-SiC(111) cannot be represented by a single Lorentzian. The broadening of the G′ band of the graphene on the 3C-SiC(111) microfacet is thus concluded to be due to presence of multiple components. The presence of multiple Lorentzian components in the G′ band suggests presence of multiple routes for this double-resonant Raman scattering process in the graphene on the 3C-SiC(111) microfacets. Several origins can be considered, which include the band splitting of graphene^42^,^43^,^44^, strain variation^28^, and carrier doping variation^47^,^48^. If strain is the cause, not only the G′ band but also the G band should show a corresponding set of multiple components^28^. The observed G band, however, shows a single component, as shown in Figure 4. The shoulder at around 1620 cm^−1^ is attributed to the so-called D′ band, induced by defects^49^. Therefore, the strain is not the cause of the multiple components of the G′ band. Carrier doping can also shift the G′ band^47^,^48^. In this case, again, the G band should also show multiple components. Moreover, the G band shift due to carrier doping should be larger than that of the G′ band^48^, which suggests appearance of even distinct multiple components in the G band. This is in contrast to the experiment. Thus, the charge density variation is excluded as the origin of the multiple components of the G′ band. We therefore suggest the band splitting of graphene as the most likely origin of the multiple components in the G′ band on the 3C-SiC(111) microfacets. This assignment is consistent with the Bernal stacking^42^,^45^ revealed by μ-LEED (Fig. 3) as well as with the presence of the buffer layer confirmed by 3D nano-ESCA (Fig. 2). There is thus a good reason to expect a semiconducting nature in the graphene on the 3C-SiC(111) microfacets.

## Discussion

This observed variation of the band dispersion is also corroborated by examining the area intensity ratio of the G′ band to the G band because this exhibits the degree of interlayer interaction^42^,^44^. The area intensity ratio is 1.2 for graphene on the 3C-SiC(111) microfacet, and 1.7 for graphene on the 3C-SiC(100) microterrace. The reduced area intensity ratio of graphene on the 3C-SiC(111) microfacet is explained mainly by a stronger interlayer interaction due to Bernal stacking of graphene on the 3C-SiC(111) microfacet, as in the case of GOS using flat Si(111) substrates. In this way, the band structure of the 3D-GOS on the microfacet and the microterrace is surely tunable microscopically by varying the graphene stacking.

In conclusion, we have succeeded in microscopically controlling the band structure of 3D-GOS through tuning of the interfaces between graphene and the 3C-SiC microfacets and microterraces with the aid of the epitaxy on the microfabricated Si(100) substrate by anisotropic wet etching, which produces Si(111) microfacets as well as Si(100) microterraces. The realization of 3D-GOS is the first step to exploiting graphene-based multifunctional integrated circuits (combining electronics and photonics) compatible with existing Si-based electronics in the next generation of devices.

## Methods

### Microscopic characterization

The spatially-resolved and angle-resolved C1s core-level photoelectron spectra are taken by using the 3D nano-ESCA system installed at the BL07LSU at SPring-8^30^,^31^,^32^, where the synchrotron radiation (SR) beam has a high energy-resolving power (*E*/Δ*E* > 10^4^). The photon energy of the SR beam for the measurement is 1000 eV. Using 3D nano-ESCA enables us to obtain a high lateral resolution (70 nm) by focusing X-rays using a Fresnel zone plate. The energy resolution of the spectrometer was set to 300 meV, and the accuracy of the angle resolution is 0.9°. The binding energy scale was calibrated by the photoelectron peaks of a gold foil (Au 4*f* 7/2). Details of the experimental setup are described in the previous reports^30^,^31^. The stacking of graphene is probed by microscopic low-energy electron diffraction (μ-LEED) using electron optics through a low-energy electron microscopy (LEEM) system installed at BL17SU at SPring-8^17^,^20^. The μ-LEED is acquired by collecting diffracted electrons from selected areas of 1-μm diameter. Raman spectra are obtained by using Raman microscopy with an excitation energy of 2.41 eV and a lateral resolution of 1 μm^17^. The X-TEM images are taken by using JEOL JEM-2010 (JEOL Ltd., Japan) with an acceleration voltage of 200,000 eV. The instrumental resolution of the transmission electron microscope is ±0.07 nm.

## Author Contributions

H.F. performed the 3D-GOS fabrication and all of the microscopic characterizations, designed the study, and participated in the writing of the manuscript. T.I. performed the 3D-GOS fabrication and all of the microscopic characterizations. Y.K. participated in fabricating the 3D-GOS. T.S. participated in the microscopic characterization using 3D nano-ESCA. N.N., K.H. and H.K. performed the microscopic characterization using 3D nano-ESCA, and participated in discussions about the content of the manuscript. M.K., T.O. and T.K. participated in the microscopic characterization using μ-LEED, and participated in discussions about the content of the manuscript. M.O. participated in the microscopic characterization using 3D nano-ESCA, and participated in discussions about the content of the manuscript. M.S. participated in discussions about the content of the manuscript.

## Supplementary Material

Supplementary Information Supplememtal Information
