# Supplementary material for: Microscopically-Tuned Band Structure of Epitaxial Graphene through Interface and Stacking Variations Using Si Substrate Microfabrication
Source: Sci Rep. 2014 Jun 6;4:5173. doi: 10.1038/srep05173 (PMC4047530; doi:10.1038/srep05173)
Supplement: Supplementary Information — Supplememtal Information [file srep05173-s1.doc]

SUPPLEMENTARY INFORMATION for

# Microscopically-Tuned Band Structure of Epitaxial Graphene through Interface and Stacking Variations using Si Substrate Microfabrication

Hirokazu Fukidome^1, 2*^, Takayuki Ide^1^, Yusuke Kawai^3^, Toshihiro Shinohara^4^, Naoka Nagamura^4, 5^, Koji Horiba^2, 4, 5^, Masato Kotsugi^6^, Takuo Ohkochi^6^, Toyohiko Kinoshita^6^, Hiroshi Kumigashira^7^, Masaharu Oshima^2, 4, 5^ & Maki Suemitsu^1, 2^

^1^Research Institute of Electrical Communication, Tohoku University, 2-1-1 Katahira, Aobaku-ku, Sendai, Miyagi 980-8577, Japan

^2^Core Research for Evolutional Science and Technology, Japan Science and Technology Agency, 5-7, Goban-cho, Chiyoda-ku, Tokyo 102-0076, Japan

^3^School of Engineering, Tohoku University, 6-6-01, Aramaki, Aoba-ku, Sendai 980-8578, Japan

^4^Department of Applied Chemistry, Graduate School of Engineering, The University of Tokyo, 7-3-1 Hongo, Tokyo 113-8656, Japan

^5^Synchrotron Radiation Research Organization, The University of Tokyo, 1-1-1 Kouto, Sayo-cho, Sayo-gun, Hyogo 679-5198, Japan

^6^JASRI/SPring-8, 1-1-1 Kouto, Sayo, Hyogo 679-5198, Japan

^7^Photon Factory, Institute of Materials Structure Science, High Energy Accelerator Research Organization, Ibaraki 305-0801, Japan

*Corresponding author: fukidome@riec.tohoku.ac.jp

Supplementary Note 1. Thickness estimation of graphene from C1s core-level photoelectron spectra

The decomposed C1s core-level spectra of graphene on the 3C-SiC microfacet are used for the thickness estimation of graphene, using the equation^1^:

$d=\lambda_{G}\cos\theta\ln\left[\frac{I_{G}}{I_{SiC}}\frac{\sigma_{SiC}}{\sigma_{G}}+1\right]$ (S1)

where *θ*, *I*_G_ and *I*_SiC_ are the photoelectron emission angle and the intensities of the decomposed peaks due to graphene and SiC, respectively. *σ*_G_ and *σ*_SiC_ are the atom densities, and their values are 3.82×10^15^ cm^-2^ and 1.22×10^15^ cm^-2^, respectively. *λ*_G_ is the inelastic electron mean free path in graphene, and is estimated as^2^:

$\lambda_{G}=\frac{538a_{A}}{E_{A}^{2}}+0.41a_{A}\sqrt{a_{A}E_{A}}$ (S2)

where *a*_A_ and *E*_A_ are the lattice constant of graphene (0.246 nm) and the kinetic energy of the photoelectrons (~712 eV), respectively. The layer numbers of graphene on the 3C-SiC(100) microfacet (2.7) and the 3C-SiC(111) microfacet (2.3) estimated using equation (S1) agree with those found in cross-sectional transmission electron microscope images (2 to 3). This confirms the validity of equation (S1).

Supplementary Note 2. Depth profile analysis of 3D-GOS by using angle-resolved spectra

The angle-resolved C1s core-level photoelectron spectra of graphene on the 3C-SiC(111) and 3C-SiC(100) microfacets were taken by using 3D nano-ESCA, as shown in Fig. S1a,b. The graphene peaks are fitted with a Doniach–Sunjic lineshape, using an asymmetry parameter of 0.1^3,4^. On the other hand, other peaks are fitted with a symmetric Voigt function^4^. A typical example of the peak decomposition is displayed in Fig. 2a,b.

The intensity ratios of SiC and peak B to graphene are then plotted against the emission angle (θ) relative to the surface normal of each facet, as displayed in Fig. 2c,d. The intensity ratio of SiC to graphene decreases with increasing emission angle. This confirms that graphene is situated on the 3C-SiC microfacets. On the other hand, the intensity ratio of peak B to the peak of graphene in the spectra of graphene on the 3C-SiC(111) microfacet does not change with the emission angle. Therefore, peak B cannot be explained only by the surface carbon contaminant. Instead, the buffer layer also contributes to peak B. Peak B should then decrease monotonically with increasing emission angle. This indicates that both the buffer layer and the surface contaminants contribute to peak B. It is demonstrated from the above depth profile analysis that the buffer layer is formed selectively between the graphene and the SiC(111) microfacet, even though surface contaminants are present on the surface.


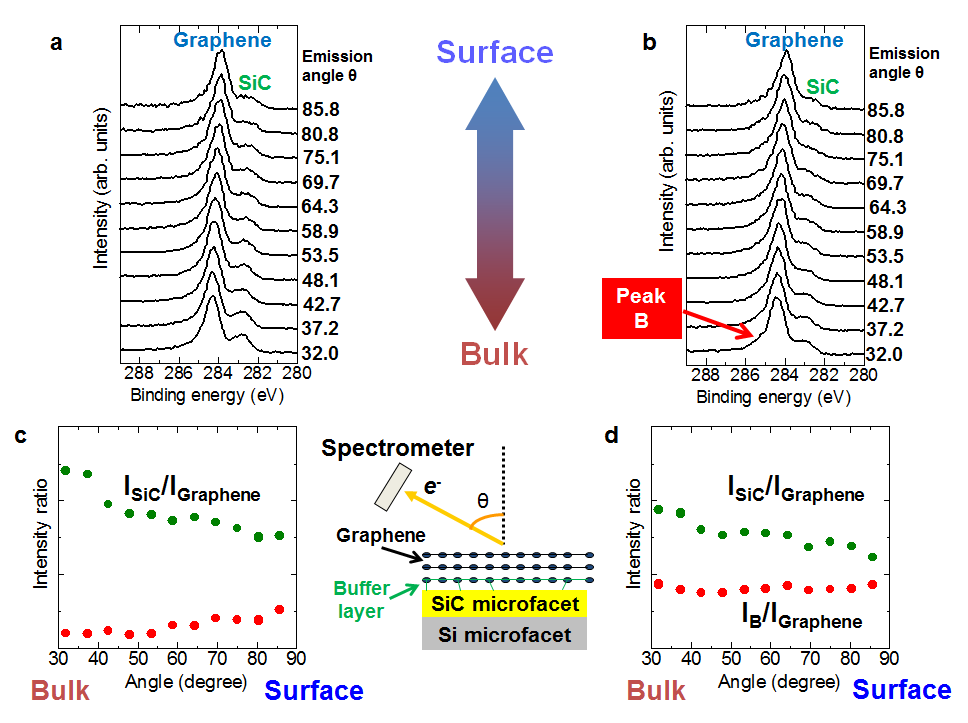


**Figure S1 | Depth profile analysis of 3D-GOS by using angle-resolved** **C1s core-level photoelectron spectra.** (**a**) Angle-resolved spectra of graphene on the 3C-SiC(100) microfacet. The emission angle is relative to the surface normal of the 3C-SiC(111) microfacet. (**b**) Angle-resolved spectra of graphene on the 3C-SiC(111) microfacet. The emission angle (θ) is relative to the surface normal of the 3C-SiC(111) microfacet. (**c**) Ratio of the intensity of the peak due to SiC to the intensity of the peak due to graphene plotted against emission angle. (**d**) Ratios of the intensities of the peak due to SiC and of peak B to the intensity of the peak due to graphene plotted against emission angle.

References

1. Horiba, K. *et al.* Scanning photoelectron microscope for nanoscale three-dimensional spatial-resolved electron spectroscopy for chemical analysis. *Rev. Sci. Instr.* **82**, 113701 (2011).
2. Seah, M. P. & Dench, W. A. Quantitative electron spectroscopy of surfaces: a standard data base for electron inelastic mean free path in solids. *Surf. Interf. Anal.* **1**, 2–11 (1979).
3. Prince, K. C. *et al*. *Phys. Rev. B* **62**, 6866–6868 (2000).
4. Nagamura, N. *et al*. Direct observation of charge transfer region at interfaces in graphene devices. Appl. Phys. Lett. **102**, 241604 (2013).
